# Supplementary material for: The dynamic adsorption affinity of ligands is a surrogate for the passivation of surface defects
Source: Nat Commun. 2024 Mar 6;15:2035. doi: 10.1038/s41467-024-46368-8 (PMC10918106; doi:10.1038/s41467-024-46368-8)
Supplement: Supplementary file 1 — Supplementary Informaion [file 41467_2024_46368_MOESM1_ESM.pdf]

Supplementary Information

The Dynamic Adsorption Affinity of Ligands is a Surrogate for the Passivation of Surface Defects

Jian Xu,[1†] Aidan Maxwell,[1†] Zhaoning Song,[2] Abdulaziz S. R. Bati,[3] Hao Chen,[1] Chongwen Li,[1] So Min Park,[1] Yanfa Yan,[2] Bin Chen,[1,3*] Edward H. Sargent[1, 3, 4*]

[1]Department of Electrical and Computer Engineering, University of Toronto, 35 St George Street, Toronto, Ontario, M5S 1A4, Canada

[2]Department of Physics and Astronomy, and Wright Center for Photovoltaics Innovation and Commercialization, University of Toledo, 2801 W. Bancroft Street, Toledo, Ohio 43606, United States

[3]Department of Chemistry, Northwestern University, 2145 Sheridan Rd, Evanston, Illinois 60208, United States

[4]Department of Electrical and Computer Engineering, Northwestern University, 2145 Sheridan Rd, Evanston, Illinois 60208, United States

[†] These authors contributed equally: Jian Xu, Aidan Maxwell

*E-mail: bin.chen@northwestern.edu, ted.sargent@utoronto.ca

Table of Contents

Supplementary Figures 1−16……………………………………………………………3−10

Supplementary Tables 1…………………………………………………………………10

Supplementary Notes 1−4…………………………………………………..…….11−12

Supplementary Methods……………………………….…………………………..….13

Supplementary References………………………………………………………........13

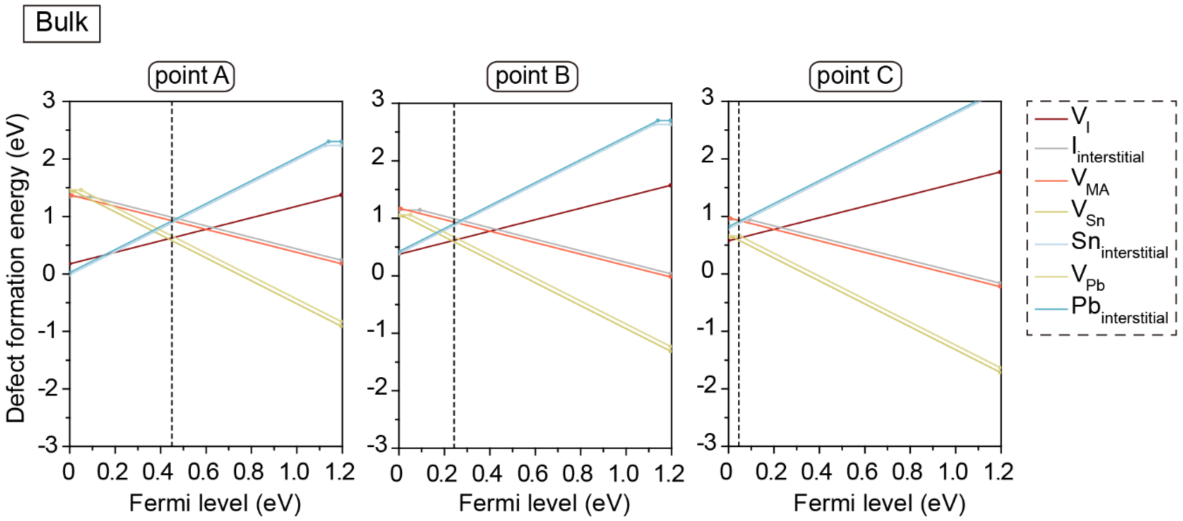

Supplementary Fig. 1 Heyd−Scuseria−Ernzerhof (HSE) including spin-orbital coupling (SOC) calculated defect formation energies of native defects in the bulk of $MAPb_{0.5}Sn_{0.5}I_3$ under the chemical potential conditions of points A, B and C. The black dashed line indicates the crossing point of the lowest-energy donor-like and acceptor-like defects.

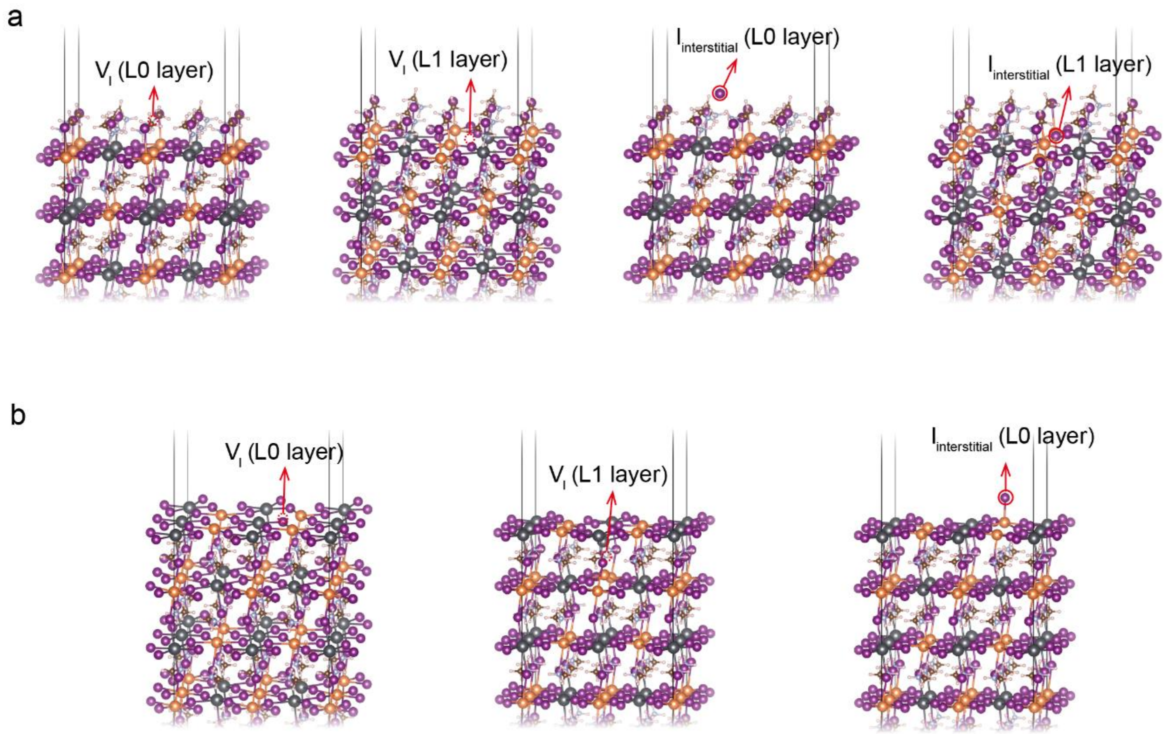

Supplementary Fig. 2 Atomic structures of defects at the (a) MAI-terminated (001) surface and (b) PbI_2-terminated (001) surface of $MAPb_{0.5}Sn_{0.5}I_3$. P atoms, lavender color; Pb atoms, grey color; Sn atoms, orange color; I atoms, purple color.

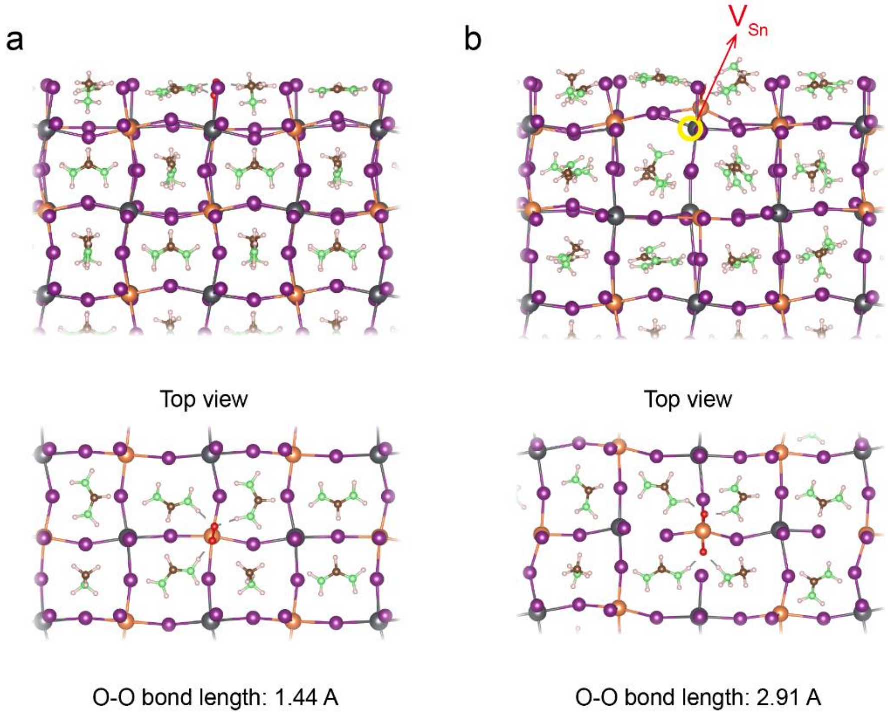

Supplementary Fig. 3 Atomic structures of $MA_{0.25}FA_{0.75}Pb_{0.5}Sn_{0.5}I_3$ (001) surface exposure to O_2^- conditions (a) in static first-principles calculations that exclude temperature effects and (b) at 300K after ~10 ps AIMD simulations.

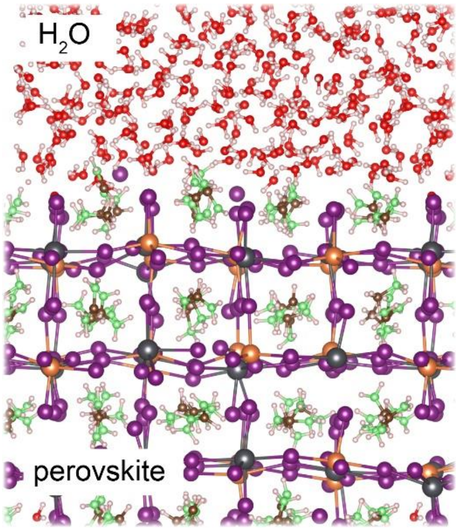

Supplementary Fig. 4 AIMD snapshots of $MA_{0.25}FA_{0.75}Pb_{0.5}Sn_{0.5}I_3$ (001) surface exposure to moisture (H_2O) conditions at a temperature of 300K. O atoms, red color; Pb atoms, grey color; Sn atoms, orange color; I atoms, purple color; N atoms, green color.

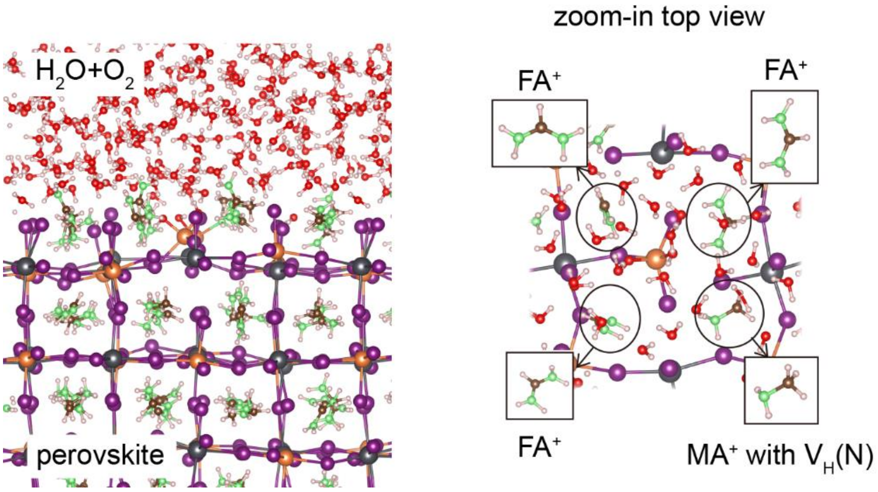

Supplementary Fig. 5 AIMD snapshots of $MA_{0.25}FA_{0.75}Pb_{0.5}Sn_{0.5}I_3$ (001) surface exposure to oxygen and moisture conditions at a temperature of 300K. O atoms, red color; Pb atoms, grey color; Sn atoms, orange color; I atoms, purple color; N atoms, green color.

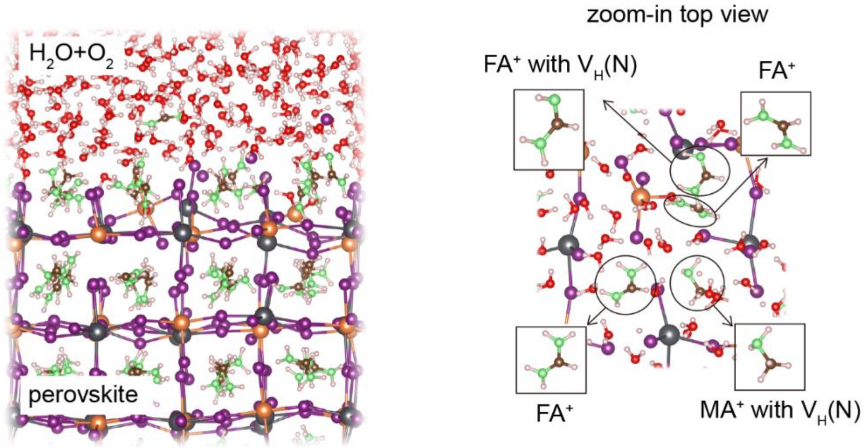

Supplementary Fig. 6 AIMD snapshots of $MA_{0.25}FA_{0.75}Pb_{0.5}Sn_{0.5}I_3$ (001) surface exposure to oxygen and moisture conditions at a temperature of 400K. O atoms, red color; Pb atoms, grey color; Sn atoms, orange color; I atoms, purple color; N atoms, green color.

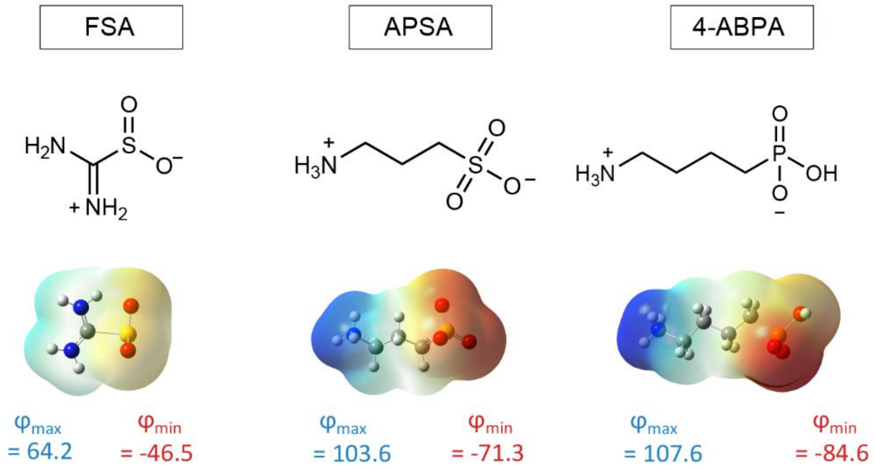

Supplementary Fig. 7 Gaussian calculated electrostatic potentials of three passivator molecules (FSA, APSA and 4-ABPA). The values of φ_{max} and φ_{min} (unit: kcal mol^{-1}) are also labeled.

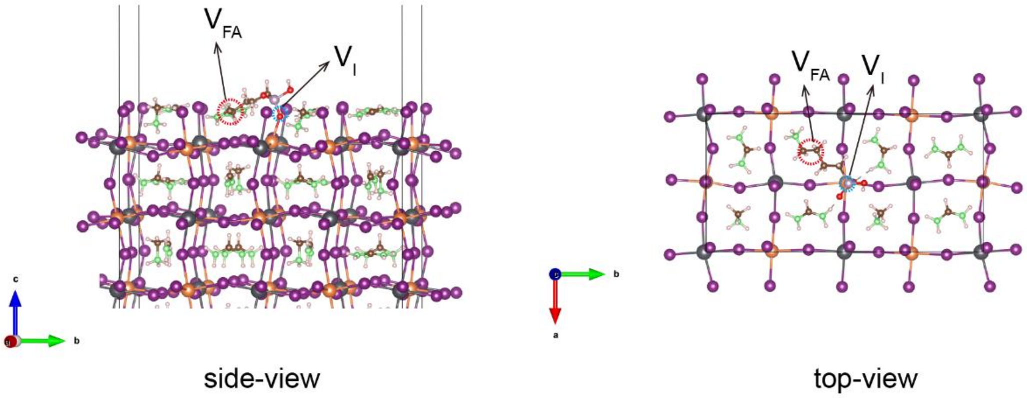

Supplementary Fig. 8 Atomic structures of 4-ABPA adsorbed perovskite surface. The perovskite composition is $MA_{0.25}FA_{0.75}Pb_{0.5}Sn_{0.5}I_3$. P atoms, lavender color; O atoms, red color; Pb atoms, grey color; Sn atoms, orange color; I atoms, purple color; N atoms, green color.

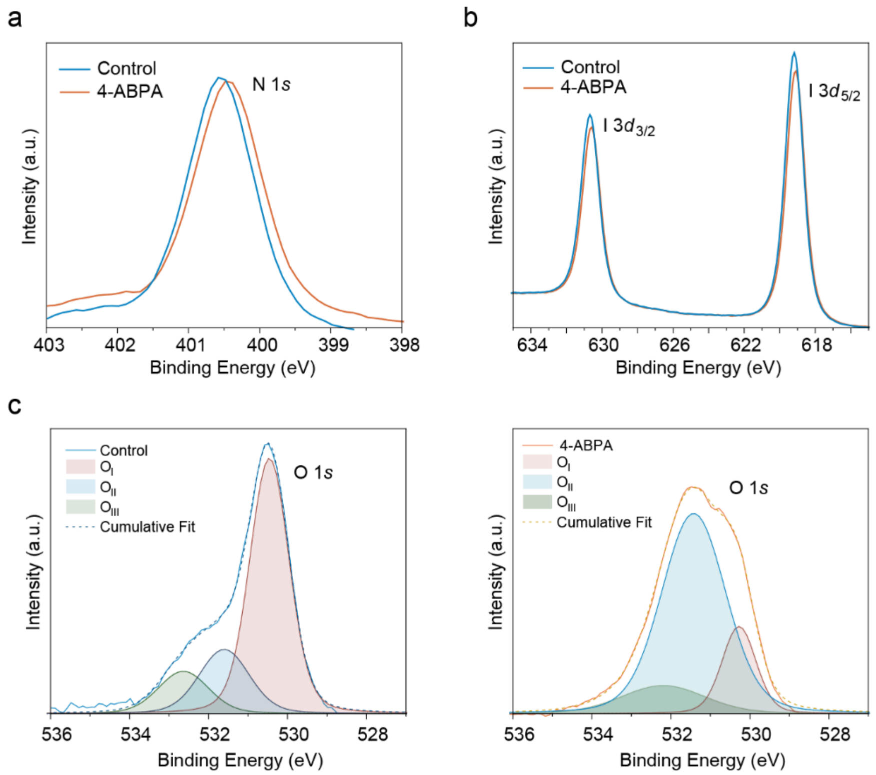

Supplementary Fig. 9 XPS spectra of **a,** N 1*s*, **b,** I 3*d* and **c,** O 1*s* for control and 4-ABPA-treated Pb-Sn perovskite films.

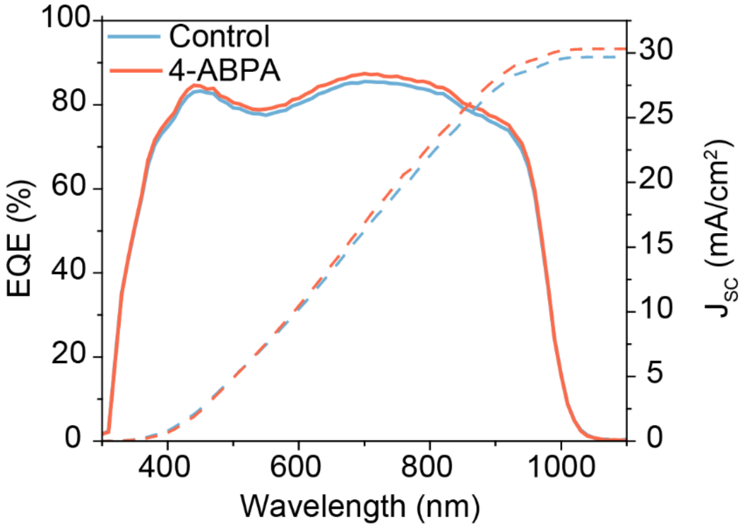

Supplementary Fig. 10 EQE of control and 4-ABPA treated Pb-Sn perovskite solar cells (PSCs).

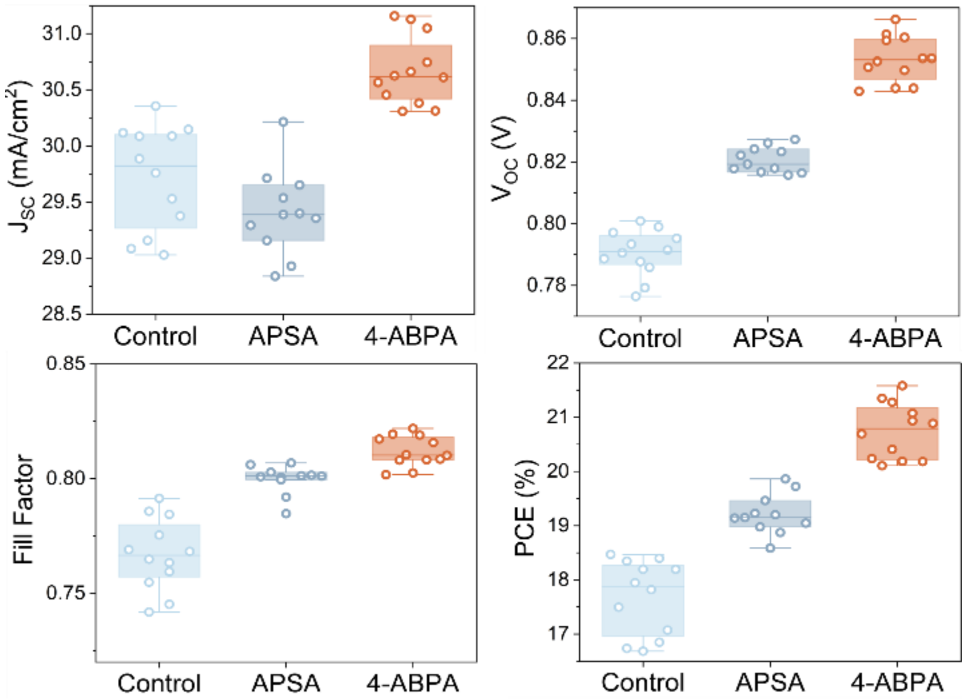

Supplementary Fig. 11 Device performance statistics for control, APSA treated and 4-ABPA treated Pb-Sn PSCs. The box plot denotes minima (bottom line), maxima (top line), median (center line), 75th (top edge of the box), 25th (bottom edge of the box) percentiles. 12 devices are tested for both control and 4-ABPA treated PSCs, while 11 devices are tested for APSA treated PSCs.

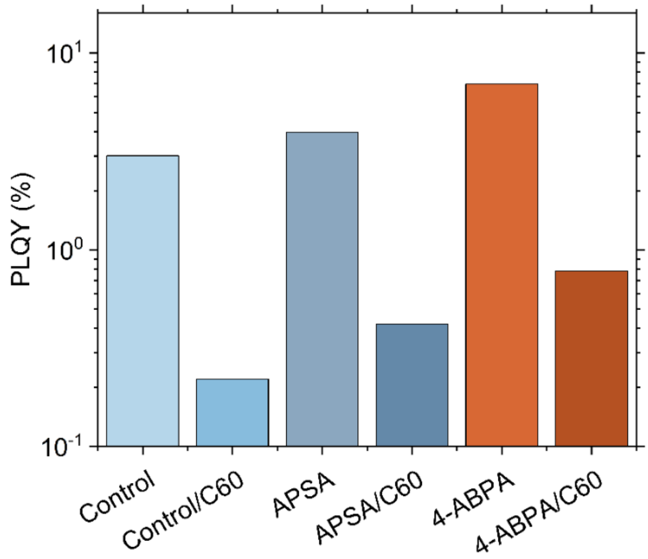

Supplementary Fig. 12 PLQY data from control, APSA-treated and 4-ABPA-treated films with and without C_{60}, on a glass substrate.

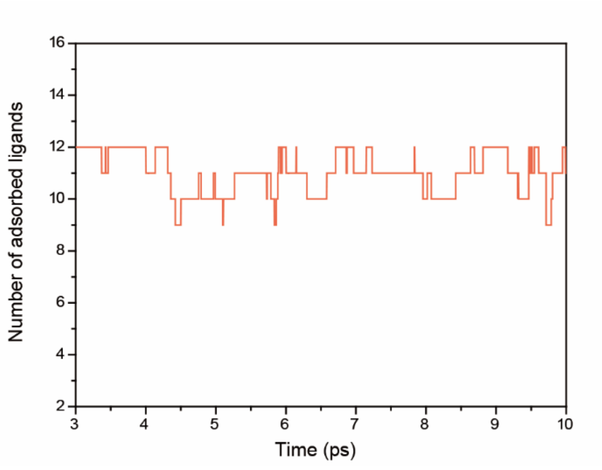

Supplementary Fig. 13 The number of adsorbed 4-ABPA ligands at the perovskite ($MA_{0.25}FA_{0.75}PbI_3$) surface exposure to oxygen and moisture conditions at a temperature of 400K during the AIMD simulation. Ligand number of 16 represents complete surface coverage within the simulation unit.

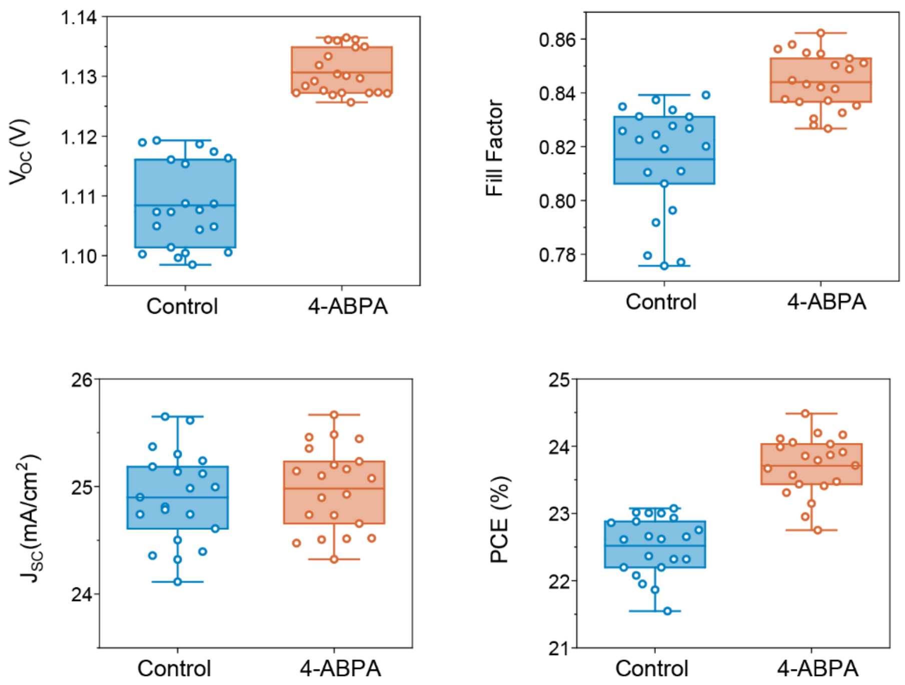

Supplementary Fig. 14 Device performance statistics of pure-Pb PSCs. The box plot denotes minima (bottom line), maxima (top line), median (center line), 75th (top edge of the box), 25th (bottom edge of the box) percentiles. 21 devices are tested for both control and 4-ABPA treated PSCs.

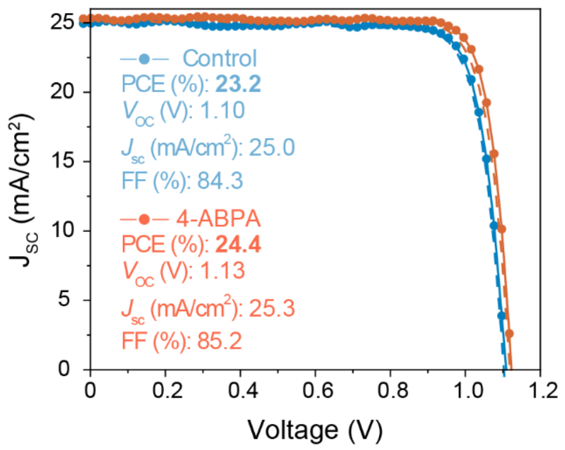

Supplementary Fig. 15 Champion forward (solid) and reverse (dashed) *J-V* curves of pure Pb (bandgap ~1.55 eV) PSCs.

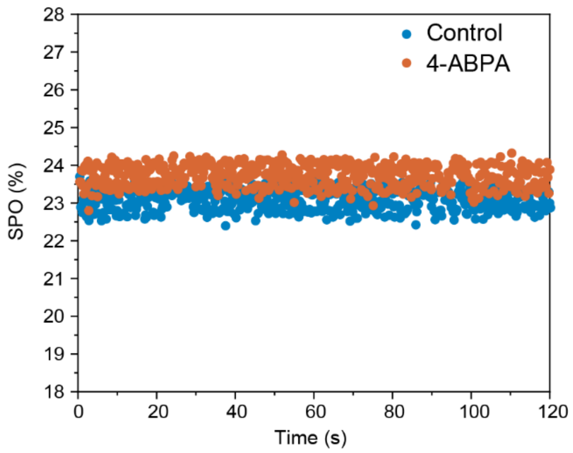

Supplementary Fig. 16 Stabilized power output (SPO) of control and 4-ABPA-treated pure Pb PSCs.

Supplementary Table 1. Comparison of the defect properties at the MAI-terminated (001) surface and PbI_2-terminated (001) surface of $MAPb_{0.5}Sn_{0.5}I_3$.

	MAI terminated surface	PbI_2 terminated surface
Defect formation energy	higher	lower
Defect properties	donors tend to be deeper	acceptors tend to be deeper
p-type feature	more *p*-type	more *n*-type

Supplementary Note 1 Charged defect calculations at the surface slabs

For charged defect calculations in three-dimensional (3D) semiconductors, a conventional jellium approach has been widely used.[1] As the size of the supercell used in calculation is increased, the formation energies of the charged defects in 3D systems gradually converge to a fixed value, whereas those in 2D systems (*e.g.*, surface slabs) diverge almost linearly: in other words, the results for 2D slabs are strongly dependent on the simulation size (*i.e.*, vacuum size),[2] which is physically illogical. Reliable correction schemes for charged defects in slab models should be included to enable more quantitative predictions of the defect formation energies;[1] there is an opportunity to increase the extent to which such corrections are implemented in simulations going forward.[3]

Supplementary Note 2 Interaction mechanism of 4-ABPA with perovskite surface

We posited that the passivator (4-ABPA) would simultaneously occupy a vacancy site for FA (V_{FA}) and an iodine vacancy site (V_I). Several bonds (interactions) can be formed as follows: 1) Hydrogen bonds between FA^+/MA^+ at the perovskite surface and the electron-rich groups (P, O) of the passivator; 2) Coordination bonds between the passivator's (P, O) groups and Sn^{2+}/Pb^{2+} at the perovskite surface; and 3) Hydrogen bonds between the NH_3^+ group of the passivator and I- at the perovskite surface.

Supplementary Note 3 The cause for the discrepancy in the relative increases of QFLS and V_{OC}

Quasi-Fermi level splitting (QFLS) is calculated by the PLQY values at various excitation light intensities:

$$QFLS = k_B T \times ln(PLQY \times S \times J_G/J_{0,rad}) \qquad \text{Supplementary Equation (1)}$$

where S is the sun-equivalent excitation intensity, (set to 1 here as an equivalent 1-sun excitation density was used) J_G is the generated current density at 1 sun (taken from device J_{SC}) and $J_{0,rad}$ the radiative recombination current in the dark (taken from the dark current value from Shockley-Queisser limit). We calculated the QFLS of the control, APSA and 4-ABPA perovskite films from the PLQY data to be 0.89 V, 0.90 V and 0.91 V respectively.

To investigate the cause for the discrepancy in the relative increases of QFLS and V_{OC}, we measured the PLQY of each of the films with a ~30 nm layer of electron transport layer (C_{60}) deposited atop the perovskite (**Supplementary Fig. 12**), since the perovskite/C_{60} interface is known to be a prominent source of non-radiative recombination in *p-i-n* devices.[4] After depositing C_{60}, the PLQY (QFLS) drops to 0.22% (0.82 V), 0.42% (0.84 V) and 0.78% (0.86 V) for control, APSA and 4-ABPA-treated stacks, respectively. It is evident that these results are more closely aligned to the device V_{OC} than the PLQY of the neat films lacking carrier transport layers. Additionally, our findings indicate that 4-ABPA not only serves as a more effective passivator of the neat perovskite, but also results in a smaller relative drop in PLQY after deposition of C_{60} than both the control film and the APSA-treated film, and therefore less interface recombination. We should note that mismatch of QFLS and VOC is very common in perovskite solar cells, as discussed in J. Warby's paper.[5]

Supplementary Note 4 Universality of 4-ABPA strategy on other perovskite compositions

We sought to probe the efficacy of our passivation strategy to other perovskite compositions beyond Pb-Sn. We began by repeating our dynamic adsorption AIMD simulations for a pure-Pb (~1.55 eV) perovskite composition (**Supplementary Fig. 13**). We found that during the last 2ps of the AIMD simulation, the number of adsorbed 4-ABPA ligands exhibited a slightly wider fluctuation range (9~10) for the pure Pb perovskite surface than for the Pb-Sn perovskite surface (11~12). This disparity suggests that the performance of Pb-Sn PSCs might experience more notable improvement than pure Pb PSCs. To test this hypothesis, we fabricated 22 control and 4-ABPA-treated devices based on a pure-Pb composition (see **Supplementary Methods**). Champion control and 4-ABPA-treated devices delivered a PCE of 23.2% and 24.4%, respectively, arising primarily from a ~20 mV increase in V_{OC} (**Fig. 4h, Supplementary Fig. 14-16**). We conclude therefore that while our strategy is applicable beyond Pb-Sn perovskite compositions, the additional benefits of suppressed Sn oxidation and enhanced dynamic adsorption offer further enhancement of photovoltaic performance.

Supplementary Methods

Pure-Pb perovskite solar cell fabrication

NiO_x nanocrystal (10 mg ml^{-1} in water and IPA mixed solution with volume ratio of 3:1) layer were first spin coated on ITO substrates at 3,000 rpm for 25 s in air without any post-treatment, then the substrates were immediately transferred to the glovebox. The NiO_x nanoparticles were synthesized via the hydrolysis reaction of nickel nitrate referring to our previous work.[6] Me-4PACz (0.3 mg ml^{-1}) in ethanol was spin coated on the NiO_x film at 3,000 rpm for 25 s and then annealed at 100 °C for 10 min. 1.5 M $Cs_{0.05}FA_{0.9}MA_{0.05}PbI_3$ perovskite precursor was prepared by dissolving the PbI_2, MAI, CsI, and FAI in DMF: DMSO (4:1 in volume) solvents, 3 mol% $MAPbCl_3$ were added into the solution to improve the quality of perovskite films. For the perovskite film fabrication, the substrate was spun at 2,000 rpm for 35 s with an acceleration of 1,000 rpm s^{-1} at first, and then at 7000 rpm for the 10 s with an acceleration of 7,000 rpm s^{-1}. In the second step, 150 μL Anisole was dropped onto the substrate during the last 5 s of the spinning. The substrate was immediately placed on a hotplate and annealed at 100 °C for 10 min. Post-treatment with 4-ABPA were carried out by spin-coating 1.5 mM 4-ABPA solutions (stirred overnight and filtered before use) in 1:1 IPA:Toluene at 4000 rpm for 25 s, followed by annealing at 100 °C for 5 min. After cooling down to room temperature, the substrates were transferred to the evaporation system, 20 nm C_{60}, 8 nm BCP and 140 nm Ag were subsequently deposited on top by thermal evaporation.

Supplementary References:

1 Walsh, A. Correcting the corrections for charged defects in crystals. *npj Comput. Mater.* **7**, 72 (2021).

2 Wang, D. *et al.* Determination of Formation and Ionization Energies of Charged Defects in Two-Dimensional Materials. *Phys. Rev. Lett.* **114**, 196801 (2015).

3 Tan, S. *et al.* Stability-limiting heterointerfaces of perovskite photovoltaics. *Nature* **605**, 268-273 (2022).

4 Warby, J. *et al.* Understanding Performance Limiting Interfacial Recombination in pin Perovskite Solar Cells. *Adv. Energy Mater.* **12**, 2103567 (2022).

5 Warby, J. *et al.* Mismatch of Quasi–Fermi Level Splitting and Voc in Perovskite Solar Cells. *Adv. Energy Mater.* **13**, 2303135 (2023).

6 Chen, H. *et al.* Quantum-size-tuned heterostructures enable efficient and stable inverted perovskite solar cells. *Nat. Photonics* **16**, 352 (2022).
