# Supplementary material for: The dynamic adsorption affinity of ligands is a surrogate for the passivation of surface defects
Source: Nat Commun. 2024 Mar 6;15:2035. doi: 10.1038/s41467-024-46368-8 (PMC10918106; doi:10.1038/s41467-024-46368-8)
Supplement: Supplementary file 3 — Description of Additional Supplementary Files [file 41467_2024_46368_MOESM3_ESM.pdf]

File Name: Supplementary Data 1

Description: Unit cell structure of $MAPb_{0.5}Sn_{0.5}I_3$ used for electronic structure calculations

File Name: Supplementary Data 2

Description: The initial and final configurations of the AIMD simulations for $FA_{0.75}MA_{0.25}Pb_{0.5}Sn_{0.5}I_3/H_2O$ interface model at 300K
